# The Impact Factor Game: It is time to find a better way to assess the scientific literature

**DOI:** 10.1371/journal.pmed.0030291

**Published:** 2006-06-06

**Authors:** The PLoS Medicine Editors

## Abstract

The *PLoS Medicine* editors argue that we need a better measure than the impact factor for assessing the biomedical literature.

We would be lying if we said that our journal's impending first impact factor is not of interest to us. What *PLoS Medicine'*s impact factor might be is certainly one of the questions that crops up most regularly in discussions with authors, and because our authors' opinions matter to us, we are obliged to take it seriously. However, for a number that is so widely used and abused, it is surprising how few people understand how a journal's impact factor is calculated, and, more importantly, just how limited it is a means of assessing the true impact of an individual publication in that journal.

A journal's impact factor is calculated from this equation:

Journal X's 2005 impact factor =
Citations in 2005 (in journals indexed by Thomson Scientific [formerly known as Thomson ISI]) to all articles published by Journal X in 2003–2004
divided by
Number of articles deemed to be “citable” by Thomson Scientific that were published in Journal X in 2003–2004

What is obvious from this equation is that the impact factor depends crucially on which article types Thomson Scientific deems as “citable”—the fewer, the better (i.e., the lower the denominator, the higher the impact factor).

Because a journal's impact factor is derived from citations to all articles in a journal, this number cannot tell us anything about the quality of any specific research article in that journal, nor of the quality of the work of any specific author. These points become particularly evident by understanding that a journal's impact factor can be substantially affected by the publication of review articles (which usually acquire more citations than research articles) or the publication of just a few very highly cited research papers.

Moreover, a journal's impact factor says nothing at all about how well read and discussed the journal is outside the core scientific community or whether it influences health policy. For a journal such as *PLoS Medicine*, which strives to make our open-access content reach the widest possible audience—such as patients, health policy makers, non-governmental organizations, and school teachers—impact factor is a poor measure of overall impact.

Despite these evident limitations, the impact factors of journals that authors publish in are very influential. Although even Thomson Scientific acknowledges that the impact factor has grown beyond its control and is being used in many inappropriate ways, the impact factors of journals have been used to decide whether or not authors get promoted, are given tenure or are offered a position in a department, or are awarded a grant. In some countries, government funding of entire institutions is dependent on the number of publications in journals with high impact factors.

Small wonder, then, that authors care so much about journals' impact factors and take them into consideration when submitting papers. Should we, as the editors of *PLoS Medicine*, also care about our impact factor and do all we can to increase it? This is not a theoretical question; it is well known that editors at many journals plan and implement strategies to massage their impact factors. Such strategies include attempting to increase the numerator in the above equation by encouraging authors to cite articles published in the journal or by publishing reviews that will garner large numbers of citations. Alternatively, editors may decrease the denominator by attempting to have whole article types removed from it (by making such articles superficially less substantial, such as by forcing authors to cut down on the number of references or removing abstracts) or by decreasing the number of research articles published. These are just a few of the many ways of “playing the impact factor game.”

One problem with this game, leaving aside the ethics of it, is that the rules are unclear—editors can, for example, try to persuade Thomson Scientific to reduce the denominator, but the company refuses to make public its process for choosing “citable” article types. Thomson Scientific, the sole arbiter of the impact factor game, is part of The Thomson Corporation, a for-profit organization that is responsible primarily to its shareholders. It has no obligation to be accountable to any of the stakeholders who care most about the impact factor—the authors and readers of scientific research. Although we have not attempted to play this game, we did, because of the value that authors place on it, attempt to understand the rules. During discussions with Thomson Scientific over which article types in *PLoS Medicine* the company deems as “citable,” it became clear that the process of determining a journal's impact factor is unscientific and arbitrary. After one in-person meeting, a telephone conversation, and a flurry of e-mail exchanges, we came to realize that Thomson Scientific has no explicit process for deciding which articles other than original research articles it deems as citable. We conclude that science is currently rated by a process that is itself unscientific, subjective, and secretive.

During the course of our discussions with Thompson Scientific, *PLoS Medicine'*s potential impact factor— *based on the same articles published in the same year*—seesawed between as much as 11 (when only research articles are entered into the denominator) to less than 3 (when almost all article types in the magazine section are included, as Thomson Scientific had initially done—wrongly, we argued, when comparing such article types with comparable ones published by other medical journals). At the time of writing this editorial, we do not know exactly where our 2005 impact factor has settled. But whatever it turns out to be, as you might guess from this editorial, we feel the time has come for the process of “deciding” a journal's impact factor to be debated openly. Something that affects so many people's careers and the future of departments and institutions cannot be kept a secret any longer.

Even more importantly, it is time to reconsider the whole process of accurately assessing an individual paper's worth not only to scientists, but also to the wider community of readers. First, although any measure of impact will remain flawed in some way, when assessing the impact of individual articles or of the papers of individuals or groups of scientists, it surely makes more sense to measure the citations specifically to those individual articles (or to papers by individuals or groups of scientists) rather than using a journal's impact factor as a proxy measure. However, it is not clear whether Thomson Scientific could measure such individual article citations accurately. Second, we urge the company to take its responsibility seriously and increase transparency and accountability. Third, we suggest that the company's staff engage in the ongoing debate among other shareholders of scientific publishing and recognize that, there are—finally—other ways of measuring impact and visibility of scholarly articles. Thomson Scientific now faces competition from organizations that have developed online tools for citation counting, such as Google Scholar and CrossRef, and this competition may help to bring about overdue change. Other measures of scientific impact may also become widely adopted, such as the usage factor, which is being promoted by the United Kingdom Serials Group ( http://www.uksg.org/rfp.pdf), or the Y factor, a combination of both the impact factor and the weighted page rank, developed by Google ( http://www.soe.ucsc.edu/~okram/papers/journal-status.pdf).

These new measures may go some way to helping assess and perhaps quantify the many roles that medical journals have, in a way that measuring citations only to research articles cannot. Magazine sections, such as those that we and other medical journals publish, not only “add value” to the research articles by interpreting them for a wider audience but have other vital roles: they may help to set agendas—by publishing policy papers or highlighting neglected health issues; give underrepresented groups, such as medical students or patient groups, a voice; or provide educational materials to physicians. Such articles will rarely be cited in indexed journals, but may be influential, for example, in changing health policy, or may be of educational value. For such articles, more relevant measures of impact may be the number of times they are downloaded, or covered in news articles, or referenced in policy documents.

Perhaps even measures such as these will become outmoded as the Internet allows for users to interact more directly with published articles. Journals have taken a step toward such a future with the publication of e-letters, and the physics preprint server arXiv.org has been promoting such interaction for many years. As more and more articles are available in full electronically and as search engines get more sophisticated at mining the Web and assessing usage, such interaction with the literature will become easier and readers will be able to judge papers for themselves rather than relying on outmoded surrogates for quality such as the impact factor. If authors are going to quote the impact factor of a journal, they should understand what it can and cannot measure. The opening up of the literature means that better ways of assessing papers and journals are coming—and we should embrace them.

